# Combined treatment using adoptive cell therapy, extended pharmacokinetic IL-2, and tumor-specific antibodies leads to cures of established B16F10 tumors and extended in vivo T cell survival

**DOI:** 10.1186/2051-1426-1-S1-P26

**Published:** 2013-11-07

**Authors:** Cary F Opel, K Dane Wittrup

**Affiliations:** 1 Chemical Engineering, MIT, Cambridge, MA, USA; 2 Biological Engineering, MIT, Cambridge, MA, USA; 3 Koch Institute, MIT, Cambridge, MA, USA

## 

**Figure 1:**
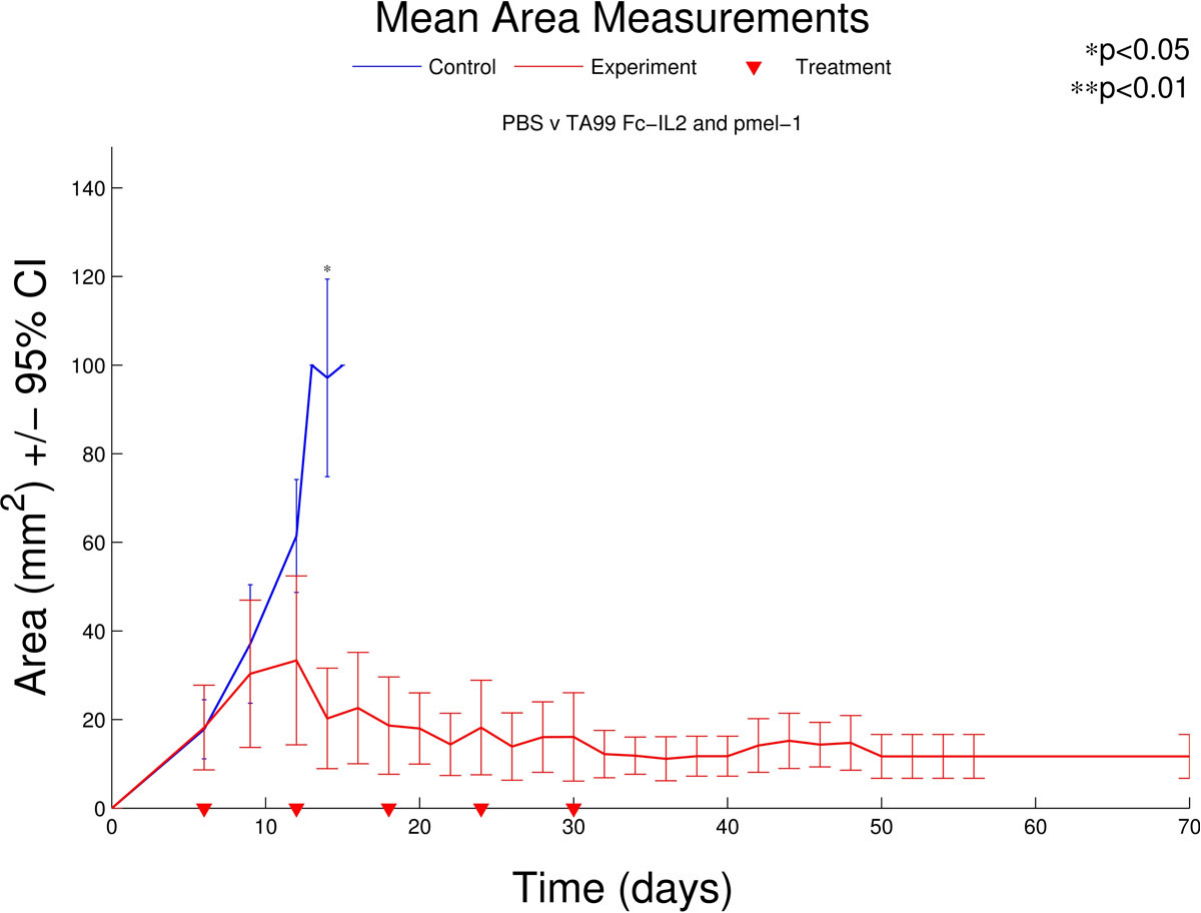
Growth curves of B16F10 tumors treated with combination immunotherapy.

IL2 is frequently given alongside adoptive cell therapy in order to enhance T cell function and survival, however, the protein has a poor pharmacokinetic profile and severe negative side effects. Fc-IL2 is a monovalent Fc fusion with IL2. The addition of the Fc domain to the cytokine significantly improved the persistence of the molecule in vivo, leading to enhanced activation of many types of immune cells, including T cells. The treatment described here is the combination of ACT, Fc-IL2, and/or antibodies targeting tumor associated antigens. The application of these three agents in preclinical experiments showed increased persistence of transferred cells, extended survival of treated animals, regression of tumor size (see Figure 1), and in some cases complete cures of established tumors with immune memory, as demonstrated by the rejection of secondary tumor challenge. Although the combination of all three agents was the most effective, the survival benefit of ACT and Fc-IL2 without antibody was significant enough to justify its use regardless of the availability of appropriate antibodies. The experimental model consisted of C57BL/6 mice subcutaneously injected with the B16F10 cell line. Following tumor establishment for 6 days, patient mice were lymphodepleted by total body irradiation and treated with a single injection of pmel-1 T cells, which express a T cell receptor (TCR) specific for B16F10 cells. In addition, over the next 24 days, 5 injections of Fc-IL2 and an antibody specific for B16F10 cells, TA99, were performed. Luciferase expressing pmel-1 cells were also injected in order to track the duration and intensity of the T cell response to the B16F10 tumors. These results show that effective adjuvants, such as Fc-IL2, have the potential to improve the clinical outcomes of adoptive cell therapy.

